# PANDEMIC-RELATED STRESS AND DAILY WELL-BEING AMONG MIDDLE-AGED AND OLDER COUPLES: RACE AND GENDER DIFFERENCES

**DOI:** 10.1093/geroni/igac059.894

**Published:** 2022-12-20

**Authors:** Kira Birditt, Angela Turkelson, Akari Oya

**Affiliations:** University of Michigan, Ann Arbor, Michigan, United States; University of Michigan, Ann Arbor, Michigan, United States; University of Michigan, Ann Arbor, Michigan, United States

## Abstract

Pandemic-related stress may have important implications for well-being among middle-aged and older couples and these effects may vary by race. Participants included 30 married/cohabiting couples (10 Black, 23 White, 2 Mixed race) ages 44 to 84 who completed baseline interviews and 5 days of ecological momentary assessment (EMAs) 6 times a day. Every three hours individuals reported how stressed they felt about COVID-19 and negative affect. Actor-partner interdependence models revealed that greater pandemic stress among Black husbands was associated with their own and their wives greater negative affect (b = 0.22, SE = 0.08, p < .01; b = 0.16, SE = 0.08, p < .05). Greater pandemic related stress among White husbands was associated with wives’ lower negative affect (b = -0.15, SE = 0.07, p < .05). Findings are consistent with structural racism theory indicating that Black individuals may be more negatively affected by pandemic-related stress than White individuals.

